# The relationship between gut microbiota and cancer immune response and immunotherapy

**DOI:** 10.3389/fimmu.2026.1833138

**Published:** 2026-06-03

**Authors:** Yue Jia, Yuechuan Liu, Jin Liu

**Affiliations:** 1 Division of Gastrointestinal & Bariatric-Metabolic Surgery, Department of General Surgery, The Second Affiliated Hospital of Dalian Medical University, Dalian, Liaoning, China; 2 Department of Education and Teaching, The First Affiliated Hospital of Dalian Medical University, Dalian, Liaoning, China; 3 Engineering Research Center for New Materials and Precision Treatment Technology of Malignant Tumors Therapy, Dalian Medical University, Dalian, Liaoning, China

**Keywords:** cancer immunotherapy, fecal microbiota transplantation, gut microbiota, ICIs, immune-related adverse events, microbial metabolites

## Abstract

The gut microbiota critically regulates cancer immunity and immunotherapy outcomes. It does so through complex, bidirectional interactions with the host immune system. Key microbial metabolites drive this process. These include short-chain fatty acids (SCFAs), tryptophan derivatives, inosine, trimethylamine N-oxide (TMAO), and bile acids. These molecules direct immune cell differentiation and activity via pattern recognition receptor signaling and epigenetic regulation. Clinical studies have linked specific microbial compositions to responses to immune checkpoint inhibitors (ICIs) across multiple cancer types. These studies also highlight a dual role of the microbiota. It can both increase therapeutic efficacy and mitigate immune-related adverse events (irAEs). Several therapeutic strategies are under active investigation. These include fecal microbiota transplantation (FMT), probiotics, prebiotics, and dietary interventions. However, challenges remain regarding engraftment, standardization, and formulation consistency. Large-scale, well-designed studies are still needed. Such studies will help establish the gut microbiota as a reliable prognostic biomarker and a viable therapeutic adjunct in cancer immunotherapy.

## Introduction

The gastrointestinal (GI) tract harbors trillions of bacteria, accounting for approximately 1–3% of the total body weight. Beyond serving as a microbial reservoir, the GI tract is the body’s largest immune organ, hosting 60–80% of all immune cells and playing a critical role in maintaining systemic immune homeostasis despite constant bacterial exposure (1). Immunotherapy has emerged as a new approach for a range of cancers, including melanoma, lung cancer, GI cancers, and hepatocellular carcinoma. However, responses to ICIs and other immunotherapies vary widely among patients, which is now attributed to differences in their gut microbiota. The intestinal microbiome shapes host immunity by releasing regulatory factors that enter the circulation and influence systemic metabolism (2). As a result, a growing number of clinicians and researchers now view the gut microbiota as both a therapeutic target and a predictive biomarker in cancer immunotherapy. Moreover, ICIs can trigger immune-related adverse events (irAEs), including colitis, hepatitis, thyroiditis, myocarditis, and type I diabetes. These complications often involve mucosal injury in the gut, breakdown of the intestinal barrier, and bacterial translocation driven by increased intestinal permeability (3). A growing body of evidence suggests that specific gut microbes may actually help manage irAEs and increase the overall efficacy of immunotherapy (4–6).

Here, we review the processes through which the gut microbiota improves the efficacy of tumor immunotherapy and reduces immune-related adverse events (irAEs) through dual mechanisms. We start by exploring the intestinal mucosal barrier, which represents innate immunity, and then analyze adaptive immunity from the impact on myeloid immune cells to that on lymphoid immune cells. We also highlight emerging technologies that hold promise for developing next-generation immunotherapies and improving the management of immune-related adverse events. This systematic exploration aims to clarify the mechanistic relationship between the gut microbiota and antitumor immune responses, thereby providing a strategic direction for future research in this field.

## Links between the gut microbiota and immunomodulation

The relationship between host immunity and the gut microbiome is bidirectional: the immune system regulates the microbial composition to maintain homeostasis, while the microbiota affects immune development and function in the meantime. This dynamic interplay has generated significant interest in whether the intestinal flora influences the response to cancer immunotherapy. The gut microbiota modulates host immunity through a range of immunomodulatory mechanisms. For instance, *Bacteroides fragilis* has been linked to mucosal dysplasia and increased polyp formation, indicating a clear role for the microbiota in shaping innate immunity in cancer (7). Gut bacteria have also been shown to disrupt neighboring mesenchymal cells that support the single-layered epithelial barrier, which is the body’s first line of immune defense against pathogens. Innate lymphocytes, which are highly enriched in the gut mucosa and associated digestive tissues, help coordinate immune balance through the secretion of immunoregulatory cytokines. Local immune responses in the gut are amplified when pattern recognition receptors (PRRs), including Toll-like receptors on intestinal epithelial cells (IECs) and other innate immune cells, detect pathogen-associated molecular patterns (PAMPs), such as lipopolysaccharides and flagellin. IgA, in turn, helps prevent bacterial adhesion to the epithelium, promotes pathogen agglutination and clearance, and modulates bacterial virulence (8–11).

Effective immunotherapy thus depends on a competent microenvironment. A growing body of evidence from large-cohort studies has established a strong association between the diversity and composition of the gut microbiome and clinical outcomes following immunotherapy across various cancer types. For instance, Chaput et al. reported that in patients with metastatic melanoma treated with ipilimumab, those whose baseline gut microbiota was enriched in *Faecalibacterium* and other *Firmicutes* species exhibited longer progression-free survival (PFS) and overall survival (OS) than those whose baseline gut microbiota was enriched in *Bacteroides* (12). Similarly, Frankel et al. reported that metastatic melanoma patients that responded to ipilimumab demonstrated enrichment of *Holdemania filiformis* and *Dorea formicigenerans* in their gut microbiota (13, 14). In a Chinese cohort of patients with colorectal cancer (CRC), the bacteria-driven activation of innate lymphocytes promoted early gut dysbiosis, compromised epithelial barrier function, and triggered innate immune signaling pathways that collectively promoted tumor development (15). This study included data from patients in multiple regions of China, but the use of perioperative antibiotics was not considered in the baseline conditions of the patients.

The gut microbiota affects not only innate immunity but also myeloid and lymphocyte immunity in the tumor microenvironment (TME) and is involved in the antitumor immune response. We elaborate on the interaction between the gut microbiota and the immune systems of myeloid or lymphoid lineages.

## The gut microbiota participates in myeloid immunity to modulate the response to ICIs

The absence of a microbiota profoundly affects the development and function of the innate immune system. Microbial communities orchestrate the differentiation and functional maturation of myeloid cells in diverse tissues and throughout their developmental trajectory. The magnitude of myeloid cell production is correlated with gut microbiome diversity and is regulated by circulating Toll-like receptor (TLR) ligands in serum (16). Microbiota-derived metabolites may similarly promote myelopoiesis (17). The immunological functions of various myeloid cell subsets are shaped by the intestinal microbiota, and increasing evidence has indicated that commensal colonization substantially remodels the host’s myeloid compartment both locally at mucosal surfaces and systemically (18, 19). *Enterotoxigenic Bacteroides fragilis* (BFT) promotes colon carcinogenesis through its toxin BFT and interleukin-17 signaling in colonic epithelial cells, leading to the recruitment and differentiation of myeloid cells into myeloid-derived suppressor cells (MDSCs) (20).

## Monocytes/macrophages

Tumor-associated macrophages (TAMs) represent a major component of the tumor microenvironment (TME) and are closely associated with organ-specific immune function and immunotherapy outcomes. In a mouse model of fatty liver disease, microbiota-derived short-chain fatty acids (SCFAs) function as signaling molecules that modify gene expression profiles in resident macrophages. The gut microbiota also produces the tryptophan metabolite indole, which activates aryl hydrocarbon receptor (AhR) signaling in TAMs, inhibiting IFNγ^+^ CD8^+^ T-cell infiltration and promoting tumor growth (21). In addition, elevated macrophage AhR expression is associated with poor immunotherapy responses in pancreatic ductal adenocarcinoma (PDAC) patients (22). Conversely, *Lactobacillus casei* combined with *Lactobacillus reuteri*, promotes macrophage polarization toward the M1 phenotype through their shared metabolite trimethylamine N-oxide (TMAO), enhancing effector T-cell responses to suppress pancreatic cancer growth and increasing immune checkpoint inhibitor (ICI) efficacy in PDAC patients (23, 24). More recently, Lee et al. demonstrated that gut microbiota-derived butyrate reduces the expression of the immunosuppressive factors PD-L1 and IL-10 in tumor-associated macrophages in gastric carcinoma patients (25). These two results show that microbial therapies enable metabolic modulation of the macrophage to enhance the efficacy of immunotherapies. However, due to the fact that these two studies were observational clinical studies and the sample sizes were limited.

## Dendritic cells

Dendritic cells (DCs) play a pivotal role in maintaining intestinal immune homeostasis by continuously surveying the gut epithelial surface for pathogens. The intestinal mucus barrier, which is composed primarily of the glycosylated mucin MUC2, actively modulates immune responses by programming intestinal DCs toward a tolerogenic phenotype (26). Tight junctions restrict paracellular permeability, and microbial metabolites such as indole improve epithelial barrier integrity by upregulating the expression of tight junction and cytoskeletal proteins. Secretory IgA and antimicrobial peptides (AMPs) further preserve mucosal barrier function. Intestinal DCs are instrumental in compartmentalizing the gut microbiota, in part by capturing bacterial antigens for presentation. Seminal work by Sivan et al. demonstrated that the commensal *Bifidobacterium* potentiates DC activation, enhancing tumor-specific CD8^+^ T-cell function and reinforcing anti-PD-L1 antibody therapeutic efficacy (27). Dendritic cells (DCs) serve as central hubs through which the gut microbiota amplifies antitumor immunity. Multiple lines of evidence indicate that distinct microbial species or their metabolites promote DC maturation and function, thereby potentiating T-cell-mediated responses against tumors. For instance, *Bacteroides fragilis* promotes DC maturation and drives Th1-polarized immune responses via IL-12 signaling, which in turn augments the antitumor effect of CTLA-4 blockade (28). This IL-12-dependent pathway appears to be involved in other contexts: vancomycin treatment has been shown to increase systemic CD8α^+^ DC abundance, supporting sustained antitumor T-cell activity in an IL-12-dependent manner following adoptive transfer. In addition to direct DC activation by intact bacteria, microbial metabolites also play a critical role. Indole-3-lactic acid derived from *Lactobacillus plantarum* suppresses colorectal cancer progression by promoting IL-12 production in DCs, thereby activating antitumor CD8^+^ T cells. Similarly, oral administration of live *Lactobacillus rhamnosus* GG improves PD-1 immunotherapy outcomes by increasing intratumoral DC and T-cell infiltration (29, 30).

Notably, ICIs themselves can actively alter the spatial distribution of gut bacteria. A recent study by Choi et al. revealed that ICIs promote the translocation of specific endogenous gut bacteria to secondary lymphoid organs, where they directly activate DCs and stimulate effector CD8^+^ T-cell responses, thereby enhancing systemic antitumor immunity (31, 32).

Mesenteric lymph nodes (mLNs), which drain the intestinal tract, serve as critical sites where commensal microbes shape adaptive immunity. Within mLNs, DCs mature following exposure to pathogen-associated molecular patterns (PAMPs). DCs acquire antigens either by extending dendrites into the gut lumen or through M cells, which are specialized intestinal epithelial cells that facilitate transcytosis. Upon activation, DCs migrate to mLNs, where they promote the differentiation of naive T cells into gut-tropic CD4^+^ T-cell subsets, including Tregs and Th17 cells (33). Collectively, these findings demonstrate that commensal microbes profoundly remodel the host myeloid cell compartment, at both the mucosal surface and systemically. Myeloid cell development and function are directed by microbially derived signals, ranging from local metabolites to circulating microbial components.

## Lymphocyte immune response between the host and the gut microbiota in cancer

The influence of the microbiota extends beyond myeloid cell development. However, the mechanisms by which commensal microbes regulate innate lymphoid cells (ILCs) appear to operate through principles that are different from those governing myeloid cells. ILCs are a recently characterized lymphocyte lineage of the innate immune system that develop normally in the absence of microbial colonization, yet their functional maturation and acquisition of tissue-specific effector functions depend on signals derived from commensal microorganisms (34). Rather than affecting lymphopoiesis, microbiota-derived signaling promotes ILC functional competence and tissue-specific adaptation.

ILCs include natural killer (NK) cells, ILC1s, ILC2s, ILC3s, and lymphoid tissue inducer (LTi) cells. These cells are predominantly localized to mucosal tissues, where they secrete cytokines that promote the expansion of beneficial bacterial strains. Recent work by Natividad et al. demonstrated that specific *Lactobacillus* strains, including *L. reuteri*, activate gut ILC3s to produce IL-22, the production of which can be restored through supplementation with tryptophan-metabolizing species. The gut microbiota also stimulates the production of IL-22 by innate immune cells such as NKT cells, γδ T cells, and macrophages via aryl hydrocarbon receptor signaling (35). Additionally, the microbiota modulates the abundance and activation status of IL-17-producing TCRγδ^+^ intraepithelial lymphocytes (IELs), contributing to host defense against pathogens and the maintenance of intestinal homeostasis (36). Nevertheless, the precise mechanisms by which the gut microbiota regulates these ILC populations remain incompletely understood and warrant further investigation. ILCs also actively shape the gut microbial composition by regulating intestinal innate immune responses, which may subsequently influence the TME. Under ILC-mediated regulation, specific commensal organisms, such as *Enterococcus hirae*, *Bacteroides fragilis*, *Bifidobacterium breve*, and *Bifidobacterium longum*, have been shown to increase cancer immunotherapy efficacy (37, 38).

Lymphocyte-mediated adaptive immune responses exhibit antigen specificity and can be modulated by the gut microbiota in mouse models. For instance, fecal bacteria from ApcMin/+ mice, particularly *Bacteroides fragilis*, are associated with mucosal dysplasia, increased polyp formation, and elevated proportions of Th17 and Th1 cells, leading to STAT3 activation in colorectal cancer (CRC) models (39). *Fusobacterium nucleatum*, which has also been linked to CRC pathogenesis, inhibits antitumor T-cell-mediated adaptive immunity (40). Microbial colonization early in life helps regulate invariant natural killer T (iNKT) cell numbers, partly through sphingolipid synthesis, thereby limiting their potential disease-promoting activities in the lung and intestinal lamina propria. Colonization by *Bacteroides fragilis*, a keystone commensal in mammals, promotes CD4^+^ T-cell differentiation and maintains the Th1/Th2 balance through its capsular polysaccharide A (PSA) (41). In the presence of activated TGF-β, this interaction drives the differentiation of CD4^+^ T cells into induced regulatory T cells, which secrete IL-10 to maintain immune homeostasis. Another landmark study in humans by Routy et al. demonstrated that the administration of *Akkermansia muciniphila* following fecal microbiota transplantation (FMT) from nonresponder patients reestablished the efficacy of PD-1 blockade (42). This effect was IL-12 dependent and occurred through the increased recruitment of CCR9^+^ CXCR3^+^ CD4^+^ T lymphocytes to tumor sites to improve ICI therapy efficacy.

**Figure 1:**
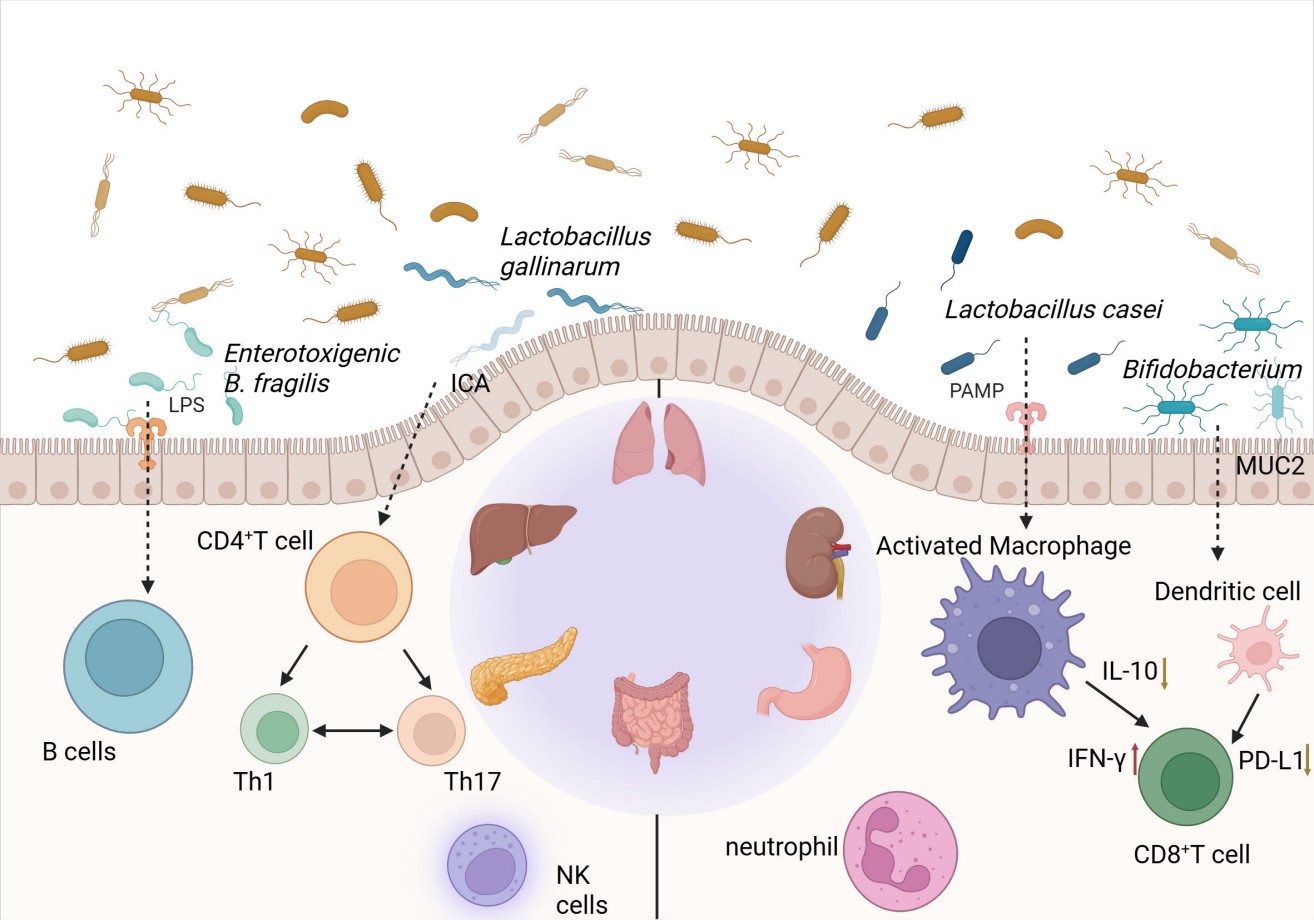
Gut microbiota interactions with intestinal immune cells. Commensal and pathogenic bacteria modulate host immunity through diverse mechanisms. *Lactobacillus casei* promotes antitumor CD8^+^ T-cell responses via PAMP receptor. *Enterotoxigenic Bacteroides fragilis* (ETBF) and its LPS modulate B-cell responses. *lipopolysaccharide gallinarum* influence CD4^+^ T-cell differentiation into Th1 and Th17 subsets. *Lactobacillus* activate macrophages. *Bifidobacterium* influence dendritic cells in the meantime. These interactions collectively shape CD8^+^ T cell and natural killer (NK) cell function activity within the intestinal microenvironment, ultimately impacting local immune homeostasis and antitumor immunity. ICA, indole-3-carboxylic acid; LPS, lipopolysaccharide. Created in https://BioRender.com.

The role of the gut microbiome extends beyond the gastrointestinal tract. In addition to directly regulating the immune response in the local mucosa through immune modulation, the gut microbiota also produces metabolites that circulate through the bloodstream and interact with immune cells throughout the body (**Figure 1**).

## Gut microbial metabolite−mediated antitumor immune responses to ICIs

Current cellular immunotherapies, including chimeric antigen receptor T-cell (CAR-T) therapy, ICIs targeting PD-1/PD-L1 and CTLA-4, natural killer (NK) cell therapy, and cancer vaccines, have demonstrated promising efficacy across multiple malignancies. These include melanoma, non-small cell lung cancer (NSCLC), gastric cancer, colorectal cancer, hepatocellular carcinoma, and other tumor types (43–47). Studies have demonstrated that specific commensals, such as *Akkermansia muciniphila*, increase the persistence and antitumor activity of CAR-T cells, improving tumor clearance (48–51). Proposed mechanisms include the production of short-chain fatty acids (SCFAs) and the modulation of T-cell activity via metabolic pathways, effectively serving as catalysts that amplify and refine immune responses.

**Figure 2:**
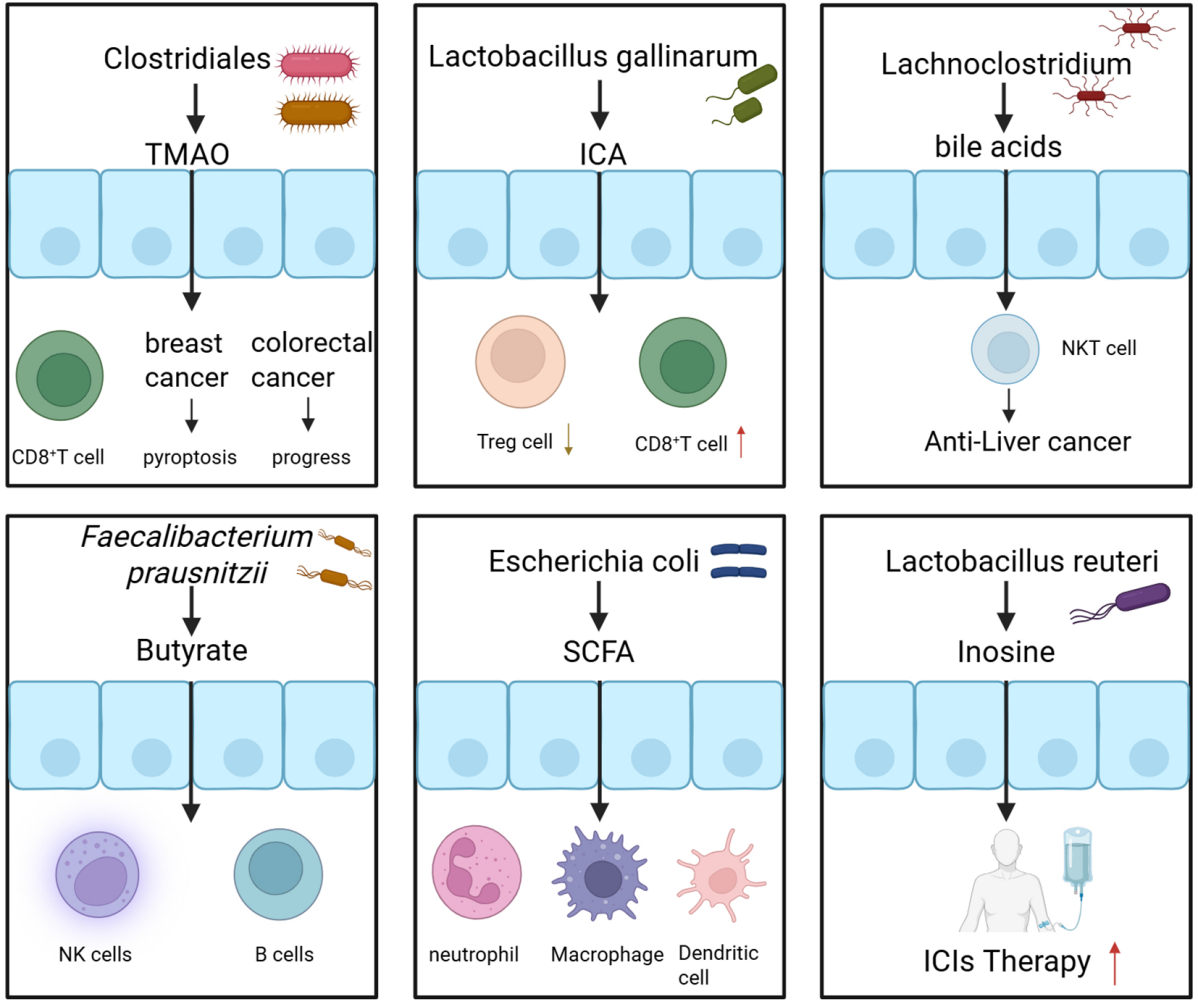
Gut microbiota-derived metabolites modulate antitumor immunity. Commensal bacteria produce diverse metabolites that shape immune responses and influence immunotherapy outcomes. *Clostridiales*-derived trimethylamine N-oxide (TMAO) enhances CD8^+^ T-cell activity against breast cancer. *Faecalibacterium prausnitzii*-generated macro influence NK cells and B cells. *Lactobacillus gallinarum* produces indole-3-carboxylic acid (ICA), which suppresses Treg differentiation while promoting CD8^+^ T-cell function. *Escherichia coli* and other SCFA-producing bacteria modulate neutrophils, macrophages, and dendritic cells. *Lachnoclostridium*-mediated bile acid metabolism enhances NKT cell antitumor activity against liver cancer. *Lactobacillus reuteri*-derived inosine potentiates ICIs therapy through adenosine receptor signaling. Created in https://BioRender.com.

Microbial metabolites serve as key mediators of host–microbiota crosstalk. Several major classes of gut microbiota-derived metabolites, including bile acids, SCFAs, and tryptophan metabolites, have been strongly implicated in the pathogenesis of immune-related disorders (**Figure 2**). Regional gradients of these metabolites, together with circulating microbial components, appear to direct the differentiation and function of immune cells. Importantly, these microbiota-driven alterations significantly influence host susceptibility to a broad spectrum of disease states.

## SCFAs

In a clinical observational study, fecal samples were collected from 74 patients with advanced gastrointestinal (GI) cancers both before and during anti-PD-1/PD-L1 treatment. Shotgun metagenomic analysis revealed that the presence of short-chain fatty acids (SCFAs), bacteria, including *Escherichia coli*, *Lactobacillus* species, and *Streptococcus* species, was positively associated with the clinical response to PD-1/PD-L1 blockade across multiple GI cancer types (52). Emerging evidence suggests a potential association between fecal SCFA concentrations and the efficacy of PD-1 inhibitors, positioning SCFAs as mechanistic links between the gut microbiota and immunotherapy outcomes. Specifically, higher levels of fecal acetic acid, propionic acid, butyric acid, and valeric acid, as well as plasma levels of isovaleric acid, were associated with significantly longer progression-free survival (PFS) in patients receiving anti-PD-1 antibody therapy (53). Compared with long-term survivors, patients with eosinophilic pneumonia (EP) exhibit reduced gut microbial metabolic activity for SCFA production and elevated p-cresol levels. In patients with non-small cell lung cancer (NSCLC) receiving anti-PD-1 immunotherapy, high fecal concentrations of acetic acid, propionic acid, butyric acid, and valeric acid and high plasma concentrations of isovaleric acid were associated with prolonged PFS (54). Notably, the results of numerous preclinical studies may provide theoretical explanations for the observed clinical efficacy of SCFA-assisted antitumor immunotherapies. Dynamic interplay exists between commensal gut bacteria and mucosal T cells, particularly regulatory T cells (Tregs). Bacterial metabolites, notably SCFAs, are essential for maintaining these local T-cell populations. The mechanism of SCFAs involves the inhibition of histone deacetylase (HDAC) activity, which implicates epigenetic regulation in this process (55). Additionally, certain gut microbes promote Treg differentiation through distinct pathways involving specific bacterial components, such as polysaccharide A, which activates Toll-like receptor (TLR) signaling in dendritic cells (56). High concentrations of SCFAs accumulate in the colon, where they lower the luminal pH, fulfill nutritional requirements, regulate microbial composition and function, and modulate immune responses. Through the engagement of G protein-coupled receptors (GPCRs) and the inhibition of HDAC activity, SCFAs influence innate immune cells, including neutrophils (via chemotactic effects), macrophages, and dendritic cells. Furthermore, SCFAs have bidirectional effects on adaptive immunity mediated by antigen-specific T cells and B cells. SCFAs also has ability to maintain intestinal barrier integrity, may serve as mediators of long-distance effects originating from the gut through the gut–lung axis. These effects can occur either directly or indirectly through stimulation of gut-associated or systemic immune pathways (57).

## Inosine

Inosine, a nucleoside composed of hypoxanthine and ribose, is an intermediate in purine metabolism. It is a natural metabolite of adenosine, and its circulating levels are influenced by dietary intake, genetic factors, and pharmaceuticals. An investigation by Zhang et al. into the metabolic profile of cancer patient plasma revealed that elevated concentrations of purine metabolites, notably inosine, were correlated with increased efficacy of ICIs (58). The results of a preclinical study may offer some explanations for this. Research has demonstrated that regulatory T-cell deficiency induces autoimmunity and causes a shift in the gut microbiota. This autoimmunity can be ameliorated by remodeling the microbiota with *Lactobacillus reuteri*, a process mediated by the metabolite inosine via its interaction with the adenosine A2A receptor (59).

## TMAO

Trimethylamine N-oxide (TMAO) is a gut microbiota–derived metabolite linked to an increased risk of cardiovascular and metabolic disorders in adults (60). In addition, TMAO, which is generated by gut bacteria of the order *Clostridiales*, has been shown to enhance antitumor immune responses driven by CD8+ T cells. This effect is achieved by triggering pyroptosis in cancer cells, thereby increasing the efficacy of immunotherapy, which was observed in a clinical observational cohort of patients with triple-negative breast cancer (n=360) using multiomics analysis (61). Additionally, in another study, TMAO was linked to carcinogenic processes in colorectal cancer patients (62).

## Tryptophan

The intestinal microbiota can directly degrade tryptophan, generating a variety of metabolites, including indole-3-lactate (ILA), indole-3-acrylate (IAC), indole-3-propionate (IPA), indole-3-aldehyde (I3A), indoleacetic acid (IAA), indole-3-acetaldehyde, and kynurenine (Kyn). Several of these compounds are also produced, in part, through the kynurenine pathway. Metabolites can be detected in the blood and fecal samples of patients, and their presence is correlated with certain diseases. Reduced levels of IPA are associated with inflammatory bowel disease, type 2 diabetes, and colorectal cancer (63). *Lactobacillus gallinarum* and its metabolite (IAC) were shown to suppress the differentiation of CD4+ regulatory T cells (Tregs) while increasing CD8+ T-cell activity. This effect is mediated through modulation of the IDO1/Kyn/AHR pathway, leading to increased efficacy of PD-1 blockade in colorectal cancer (CRC) patients (64).

## Bile acids

Modulation of host bile acid metabolism by the intestinal microbiota may play a role in the regulation of cancer immunotherapy. Interactions between the fecal microbiota and bile acids have been linked to treatment outcomes in patients with unresectable hepatocellular carcinoma (HCC) receiving immune checkpoint inhibitor (ICI) therapy (65). This study prospectively enrolled patients with unresectable hepatocellular carcinoma (uHCC) receiving ICI treatment between May 2018 and February 2020. Fecal samples were collected prior to treatment. The analysis included 20 patients with radiology-confirmed objective responses (OR) and 21 randomly selected patients with progressive disease (PD). Starting in March 2020, a validation cohort of 33 consecutive Child-Pugh-A patients was recruited. Fecal samples from 17 healthy volunteers were also collected for baseline microbial comparison. An increase in bile acids resulting from post-antibiotic ileal dysbiosis downregulates MAdCAM-1. This reduction, in turn, initiates the migration of immunosuppressive T cells from gut-associated lymphoid tissues to tumors (66). Specifically, the levels of ursodeoxycholic acid and ursocholic acid markedly increased in the feces of patients who experienced objective clinical responses, a finding closely associated with the relative abundance of *Lachnoclostridium* and a reduction in the abundance of *Prevotella*. Additionally, the preclinical evidences showed that the gut microbiome utilizes bile acids as signaling molecules to influence the chemokine-mediated accumulation of natural killer T cells in the liver, which enhances antitumor immunity against both primary and metastatic liver tumors (67).

## Therapeutic strategies utilizing the gut microbiome combined with ICIs

The mechanistic insights discussed above have laid a foundation for clinical translation. Researchers have begun to develop therapeutic strategies that intentionally harness the gut microbiome. These approaches include the use of probiotics, which are live beneficial bacteria and prebiotics. The dietary substrates promote favorable microbial populations. Another strategy is fecal microbiota transplantation (FMT), in which entire functional microbial ecosystems are transferred to recipients from healthy donors. When combined with immune checkpoint blockade, these interventions aim to enhance antitumor immune responses and reduce immune-related adverse events.

## Probiotics

Probiotics are live microorganisms that provide a health benefit to the host. Historically, early-phase clinical trials involving cancer patients have focused primarily on assessing how these beneficial microbes could alter the composition of the gut microbiota or modulate the body’s antitumor immune responses. With a well-documented history of safety, *Lactobacillus* and *Bifidobacterium* are classified as probiotics and have been granted generally recognized as safe status (68). Additionally, several gut-resident microbes, such as *Bifidobacterium*, *Akkermansia*, *Enterococcus*, and *Faecalibacterium*, play critical roles as immune adjuvants, significantly improving the therapeutic outcomes of patients receiving immune checkpoint blockade (69–71). A growing body of evidence suggests that the antitumor response to PD-1 blockade can be enhanced by *Lactobacillus* species, an effect mediated through the expansion of commensal microbes and subsequent alterations to the functional profile of the gut metagenome (72). In the context of hepatocellular carcinoma (HCC), the administration of *Lactobacillus acidophilus* increases the abundance of beneficial symbionts in the fecal microbiota and markedly inhibits the progression of MASLD-HCC (73). Spencer et al. conducted a parallel preclinical investigation to evaluate the effects of a commercially available probiotic supplement in the context of melanoma. Their findings revealed that mice administered probiotics exhibited a reduced therapeutic response to anti-PD-1-based treatment, accompanied by a reduced frequency of interferon γ-positive cytotoxic T cells within the tumor microenvironment (74). Moreover, although PD-1 inhibitors have demonstrated considerable therapeutic efficacy in oncology, their use is frequently accompanied by immune-related adverse events (irAEs), including colitis and hepatitis (75). Notably, *Lactobacillus rhamnosus* has been reported to regulate inflammatory signaling pathways in both the intestinal tract and liver in murine models of HCC, indicating its potential utility in alleviating such irAEs while concurrently improving therapeutic responses (76). Despite increasing interest in the use of probiotics during immunotherapy, controversy persists over the effectiveness of over-the-counter probiotic products sold as dietary supplements. Key obstacles include the absence of standardization in strain composition, dosing, and quality assurance. Moreover, clinical results involving probiotic preparations in the context of cancer immunotherapy have shown considerable heterogeneity, with certain investigations revealing marginal or no therapeutic benefits. These issues highlight the need for additional studies aimed at establishing standardized formulations and clarifying their underlying mechanisms, which would promote reproducible and robust treatment outcomes.

## Prebiotics

A prebiotic is a substrate that confers health benefits by selectively stimulating beneficial host microorganisms. Emerging evidence underscores the crucial involvement of prebiotics in immune modulation, gut barrier integrity preservation, and the regulation of metabolic function (62, 77, 78). Prebiotics are employed to offer a selective advantage to beneficial microorganisms, as opposed to the direct administration of probiotics. Notably, compared with the administration of live bacteria or complex bacterial transplants, the oral delivery of microbiome-derived compounds, such as bacteriophages and bacterial metabolites, may offer a more feasible and targeted approach (79). Extracted from plant cell walls, the soluble fiber pectin is readily fermented by the gut microbiota and acts as a primary substrate that drives the production of numerous metabolic byproducts. Specifically, prebiotics may augment the immunomodulatory effects of ICIs by modulating the levels of SCFAs, which subsequently increase systemic memory T-cell activity and facilitate T-cell recruitment and activation within the tumor microenvironment (80, 81).

## Antibiotics and dietary intervention

A diverse range of environmental exposures, including diet and pharmaceutical intake, actively shape the human gut microbial community. The administration of antibiotics is known to reduce microbial diversity and consequently modify the composition of the microbiota. Although antibiotics are frequently coadministered with immunotherapy to manage or prevent severe infections, accumulating evidence from preclinical studies indicates that antibiotic use may compromise the efficacy of immunotherapeutic interventions. In line with this, Kim et al. reported that prior exposure to antibiotics (pATB) is correlated with significantly reduced progression-free survival (PFS) and overall survival (OS) across multiple cohorts of patients with advanced gastric cancer (GC) receiving PD-1 inhibitor therapy. When treatment responses were analyzed, the objective response rate (11.8% vs. 1.5%) and the disease control rate (52.9% vs. 16.4%) were significantly greater in the non-pATB group than in the pATB group. In addition, pATB administration was associated with decreased PFS (hazard ratio [HR] = 2.897; 95% confidence interval [CI] = 2.043–4.109) and OS (HR = 2.294; 95% CI = 1.622–3.242) among patients treated with PD-1 inhibitors (82). Adherence to healthful dietary patterns and the intake of specific nutritional elements can stimulate the enrichment of a beneficial intestinal microbiota, consequently aiding in cancer prevention and the promotion of well-being. The link between diet and the gut microbial community affects tumorigenesis and cancer progression through the modulation of host metabolic and immune pathways. This dynamic interplay also has the potential to sculpt the landscape of cancer immunosurveillance and influence therapeutic outcomes with immunomodulatory agents. Simpson et al. investigated how diet shapes the impact of the gut microbiome on cancer immunotherapy outcomes and reported that inadequate baseline consumption of fiber and omega-3 fatty acids and insufficient levels in the peripheral circulation were associated with a reduced therapeutic response (83). For example, the ketogenic diet, characterized by high fat intake and low intake of carbohydrates and proteins, mitigates lactate-mediated immunosuppression in tumors and reshapes their metabolic processes (84). Additionally, analysis of fecal samples indicated that a ketogenic diet induced remodeling of the gut microbiota, resulting in the proliferation of CXCR3^+^ T cells and the suppression of IFN γ-mediated PD-L1 expression on myeloid cells (85). Moreover, a ketogenic diet inhibited hepatocellular carcinoma (HCC) progression through the upregulation of HMGCS2 protein expression. Notably, overexpression of HMGCS2 reduces CXCL12 expression by downregulating HDAC1-dependent KLF5 expression, thereby mitigating the immunosuppressive TME (86). This process promotes the infiltration of natural killer (NK) cells and cytotoxic T lymphocytes, thereby increasing the effectiveness of anti-PD-1 antibody treatment in colorectal cancer patients. These discoveries offer a robust theoretical basis for continued exploration into the potential of a ketogenic diet to increase the response to anti-PD-1 antibody therapy (87).

## Fecal microbial transplantation

FMT was originally developed as a treatment for recurrent *Clostridium difficile* infection that did not respond to standard therapy (88). In recent years, increasing evidence has shown that FMT can increase the antitumor activity of ICIs and reverse resistance to immunotherapy. This hypothesis is currently being tested in several clinical trials in which the combination of FMT with ICI treatment is being evaluated. Elkrief et al. reported that fecal microbiota transplantation (FMT) from patients with non-small cell lung cancer (NSCLC) who responded to immunotherapy increased the efficacy of PD-1 blockade in germ-free or antibiotic-treated mice, whereas FMT from nonresponders did not yield the same benefit (89). In a phase I clinical trial (NCT03353402), ten melanoma patients unresponsive to PD-1 blockade received FMT followed by reinduction of anti-PD-1 therapy (90). Among them, three patients exhibited tumor volume reduction, including two partial responses (PR) and one complete response (CR). In another concurrent phase I trial (NCT03341143), fifteen anti-PD-1-resistant melanoma patients were treated with FMT plus pembrolizumab. Three patients achieved PR, and three others had stable disease (SD) lasting more than 12 months (91). A novel strategy to reverse resistance to ICI-based immunotherapy and reduce the incidence of irAEs involves the application of FMT (92). Total of 20 previously untreated patients with advanced melanoma were enrolled, and the primary endpoint was safety. No grade 3 adverse events were attributed to FMT alone. Five patients (25%) experienced grade 3 immune-related adverse events resulting from the combination therapy. These findings indicate that FMT represents a novel strategy to reverse resistance to ICI immunotherapy and to reduce immune-related adverse events (irAEs). Ongoing clinical trials are actively investigating whether fecal microbiota transplantation (FMT) can increase the response to immunotherapy in cancer patients experiencing tumor recurrence or therapeutic resistance (**Table 1**). These studies focus on diverse cancers, such as melanoma, and those of the gastrointestinal tract and prostate. Baruch and colleagues reported that this combined approach was not only safe but also linked to positive changes in immune cell profiles and gene activity within both the gut lining and the tumor site (93). Nonetheless, the application of FMT in clinical settings faces certain barriers. Although hailed as a revolutionary intervention demonstrating remarkable efficacy against refractory *Clostridium difficile* infections, inflammatory bowel disease (IBD), and even neurodegenerative disorders such as Parkinson’s disease and depression, the core challenge lies not in the quantity of microbes administered but in their ability to engraft, persist, and perform their intended functions within the host. A population-based study revealed that among immunocompromised patients, the microbial engraftment rate was below 40%, often resulting in disease relapse (94). In contrast, individuals with preserved T-cell functionality, intact intestinal epithelial barriers, and robust stem cell activity experienced significantly prolonged therapeutic benefits from FMT. In essence, if the structural and immunological infrastructure of the gut is compromised, even the most potent microbial consortium will fail to take root. A sluggish immune system offers a cold reception to the incoming bacteria, while the depletion of stem cell reserves leaves microbial signals unanswered.

**Table 1:** Clinical trials of FMT modulate the efficacy and AEs of ICI.

NCT number	Cancer types	n	Intervention	Outcome(s)	Stage
Modulation of the gut microbiome to improve ICI efficacy					
NCT04758507	Renal cell carcinoma	50	Donor FMT + ICI versus Placebo FMT+ICI	PFS, ORR, AEs	Phase 1-2
NCT07432984	Non-Small Cell Lung Cancer	15	FMT+ Tislelizumab	AEs, ORR, PFS	Phase 2
NCT05286294	Squamous Cell Carcinoma	20	FMT+ ICI	ORR, OS	Phase 2
NCT05690048	Hepatocellular Carcinoma	48	FMT+ Atezolizumab + Bevacizumab	AEs	Phase 2
NCT04264975	Solid Carcinoma	60	FMT+ ICI	ORR	NA
NCT05533983	Solid Carcinoma	50	FMT+ Nivolumab	ORR	NA
NCT06403111	Non-Small Cell Lung Cancer	62	FMT+ Chemotherapy+ Immunotherapy	AEs, ORR	Phase 2
NCT05669846	Non-Small Cell Lung Cancer	26	FMT+ Pembrolizumab	ORR, PFS	Phase 2
NCT06643533	Hepatocellular Carcinoma	15	FMT+ Sintilimab	ORR, PFS, OS	NA
NCT06486220	Nasopharyngeal Carcinoma	96	FMT+ Anti-PD-1	PFS, OS	Phase3
NCT07463248	Hepatocellular Carcinoma	64	FMT+ Tislelizumab	OS, ORR, AEs	Phase 2
NCT04521075	Melanoma	42	FMT+ Nivolumab	PFS, OS	Phase 1-2
NCT07247786	Non-Small Cell Lung Cancer	68	FMT+ ICI	ORR, PFS, OS	Phase 2
NCT05008861	Non-Small Cell Lung Cancer	20	FMT+ Anti-PD-1	AEs, ORR	Phase 1
Modulation of the gut microbiome to prevent ICI-related AEs					
NCT03819296	Melanoma+ Lung Cancer	800	FMT+ ICI	ICl-related colitis	Phase 1
NCT04163289	Renal cell carcinoma	20	FMT + lpilimumab/Nivolumab	ICl-related colitis	Phase 1
NCT04038619	Renal cell carcinoma	40	FMT+ ICI+ Loperamide	ICl-related diarrhea	Phase 1
NCT04883762	Solid tumors	10	FMT+ ICI	ICl-related diarrhea	Phase 1

www.clinicaltrials.gov. AE, adverse events; ICI, immune checkpoint inhibitor; n, number of patients; NA, not applicable; ORR, objective responses rate; PFS, progression-free survival; OS, overall survival.

## Modulation of the gut microbiome to prevent ICI-related adverse events

The advent of ICIs was a transformative advancement in oncology. Nevertheless, their widespread use is limited by immune-related adverse events (irAEs) (95). These adverse effects have the potential to involve nearly any organ system, with the skin, gastrointestinal tract, liver, and endocrine glands being most frequently affected. Evidence indicates that irAEs result from the off-target effects of an excessively activated immune system on healthy tissues. In patients receiving ICI monotherapy, the incidence of irAEs can reach as high as 50%. Clinically, irAEs induced by ICIs often resemble autoimmune or inflammatory conditions affecting the same organs (96). For instance, ICI-mediated colitis presents similarly to colitis associated with ulcerative colitis or Crohn’s disease. At present, there are no established, targeted therapies specifically for irAEs. However, successful treatment of corticosteroid-resistant ICI-induced colitis through FMT has been reported. High-throughput metagenomic sequencing of fecal samples from patients receiving ICI therapy revealed distinct gut microbial profiles associated with immune-related adverse events (irAEs) (97). Studies consistently report an increased abundance of potentially harmful bacteria, such as *Bacteroides intestinalis*, along with a reduction in potentially beneficial microbes, including various *Bacteroides* species, *Ruminococcus*, *Bifidobacterium*, *Faecalibacterium prausnitzii*, and *Lactobacillus*, at baseline in individuals who later develop irAEs (98). The protective effects of *Bifidobacterium*, *F. prausnitzii*, and *Lactobacillus* have been validated in mouse models of ICI-induced irAEs. The interplay between the gut microbiota and immune cells is well established in both health and disease, including cancer, and plays a significant role in shaping responses to ICI treatment (99).

## Discussion

**Figure 3:**
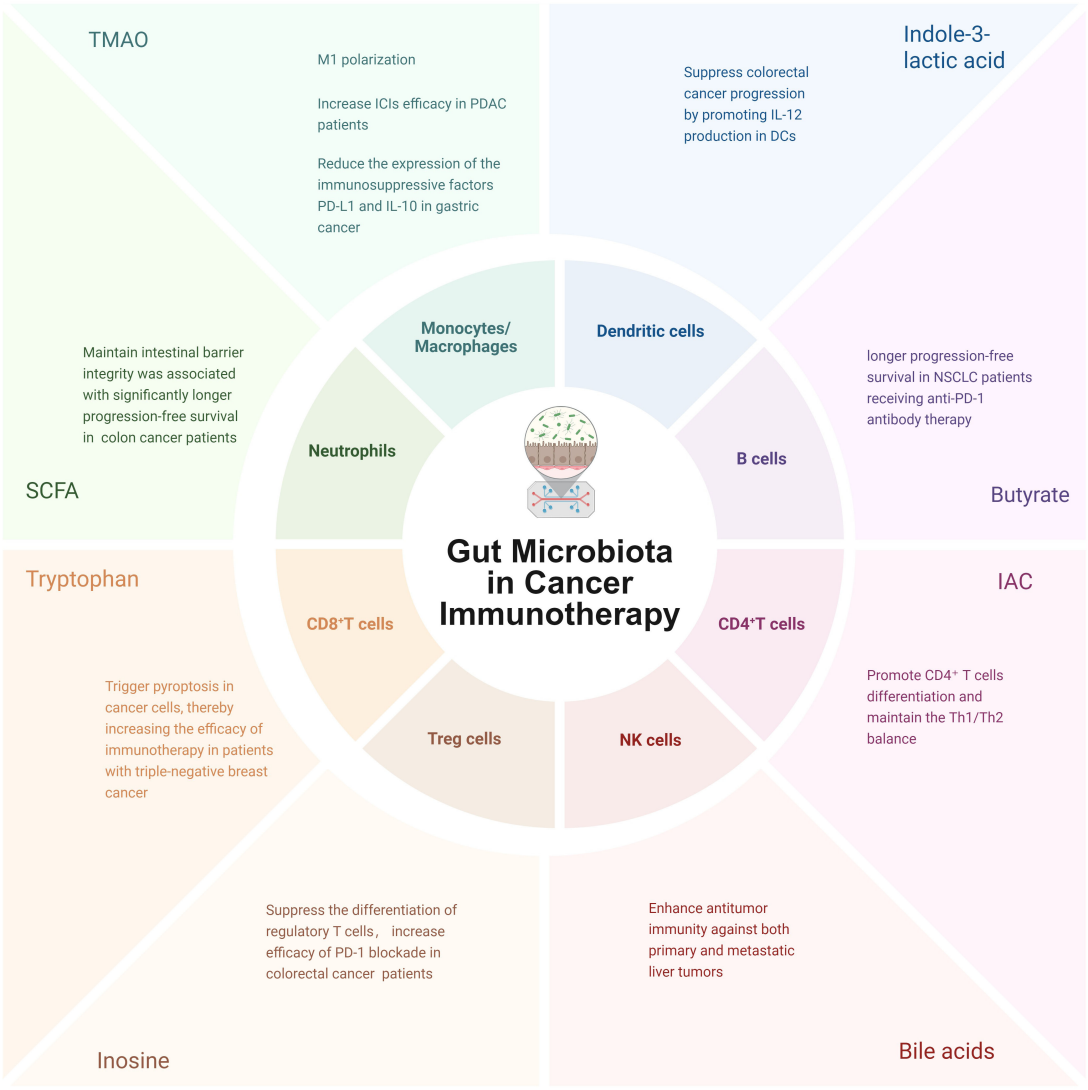
The relationship between gut microbiota and cancer immunotherapy. Created in https://BioRender.com. TMAO, trimethylamine N-oxide; ICA, indole-3-carboxylic acid.

The gut microbiome plays a crucial role in modulating cancer immunity and influencing the efficacy of immunotherapy (**Figure 3**). Collectively, these modifications reshape the immune landscape within the tumor microenvironment and ultimately enhance responsiveness to immune checkpoint inhibition. In this context, microbiome-based precision medicine has emerged as a promising therapeutic strategy in oncology. This approach aims to offer a more targeted and safer modality of microbial intervention that facilitates immune-mediated tumor clearance (100–102).

Numerous related strategies are currently under evaluation in clinical trials, including the use of highly immunogenic live commensals, antibiotics that selectively target detrimental bacterial populations, genetically engineered vaccines incorporating cancer epitopes along with microbial adjuvants, monoclonal microbial products derived from specific strains, FMT, prebiotics, probiotics, immunostimulatory dietary formulations, and adjuvants designed to potentiate the antitumor efficacy of bacterial agents (103, 104).

## Unresolved issues and contradictory findings

Despite growing evidence linking the gut microbiota to immunotherapy outcomes, several fundamental issues remain unknown. First, whether specific microbial signatures are universally predictive across cancer types or are tumor-specific remains unclear. For example, while *Akkermansia muciniphila* has been associated with favorable responses in patients with non-small cell lung cancer or renal cell carcinoma, its role in other malignancies is less consistent. Second, contradictory findings have been reported regarding the roles of SCFAs. Some studies have noted that SCFAs improve CD8^+^ T-cell effector function and ICIs efficacy, whereas others suggest that high systemic levels of SCFAs may impair antitumor immunity by suppressing T-cell trafficking. Third, the temporal dynamics of changes in the microbiota during ICIs treatment remain poorly characterized. Most studies have focused on baseline microbiome composition, but whether dynamic shifts that occur during treatment are better predictors of the outcome than static measurements are is unknown.

## Translational challenges

Several barriers hinder the clinical translation of microbiome-based interventions. One major challenge is the lack of standardized protocols for microbial profiling, including differences in sample collection, DNA extraction, sequencing platforms, and bioinformatic pipelines. These technical variations lead to poor reproducibility across studies. Another challenge is the inconsistent engraftment of transplanted microbes following FMT. Successful engraftment depends on recipient factors such as baseline microbial diversity, antibiotic exposure, diet, and immune status. Additionally, regulatory and safety concerns remain, including the determination of optimal dosing parameters (such as the frequency of administration, route of delivery, use of single versus pooled donor material, aerobic versus anaerobic processing, and product formulation), the potential transmission of antibiotic-resistant pathogens and the long-term consequences of manipulating the gut ecosystem in cancer patients. The role of recipient characteristics in determining FMT success also requires further investigation. Owing to the inherent heterogeneity in current FMT protocols, comparing outcomes across trials is highly challenging, even within the same clinical indication. Furthermore, how to predict which patients are likely to respond to FMT intervention remains unclear.

Importantly, the role of potential confounding factors, such as diet, environmental factors, ethnicity, comorbidities, and medications other than antibiotics, has not been systematically investigated in most clinical trials to date.

## Future directions: multiomics and precision microbiome modulation

To address the above challenges, future research should adopt multiomics approaches that integrate metagenomics, metabolomics, transcriptomics, and proteomics. Such integrative analyses can progress beyond taxonomic correlations to identify causal microbial functions and metabolites. For instance, combining shotgun metagenomics with untargeted metabolomics can link specific bacterial genes to immunomodulatory metabolites. Single-cell RNA sequencing of tumor and immune cells can reveal how microbiota-derived signals influence the tumor microenvironment at cellular resolution.

Precision microbiome modulation represents another promising direction. Rather than using unselected FMT or broad-spectrum probiotics, future strategies should aim to administer defined bacterial consortia or engineered microbial strains that produce specific immunomodulatory metabolites. Synthetic biology approaches could enable the development of edited microbes that deliver therapeutic payloads only within the tumor microenvironment or in response to specific signals. Furthermore, personalized microbiome interventions based on baseline profiling of a patient’s gut microbiota, diet, and immune status may improve outcomes.

Well-designed, large-scale prospective studies with standardized protocols are urgently needed to establish the gut microbiota as a reliable prognostic biomarker and a viable therapeutic adjunct in cancer immunotherapy. Collaborative efforts across academic centers and industry will be essential to overcome current translational barriers and realize the promise of microbiome-based precision oncology therapy.
